# Supplementary material for: The prion-like domain of Drosophila Imp promotes axonal transport of RNP granules in vivo
Source: Nat Commun. 2019 Jun 13;10:2593. doi: 10.1038/s41467-019-10554-w (PMC6565635; doi:10.1038/s41467-019-10554-w)
Supplement: Supplementary file 1 — Supplementary Information [file 41467_2019_10554_MOESM1_ESM.pdf]

Supplementary Information

The prion-like domain of *Drosophila* Imp promotes axonal transport of RNP granules *in vivo*.

Vijayakumar et al.

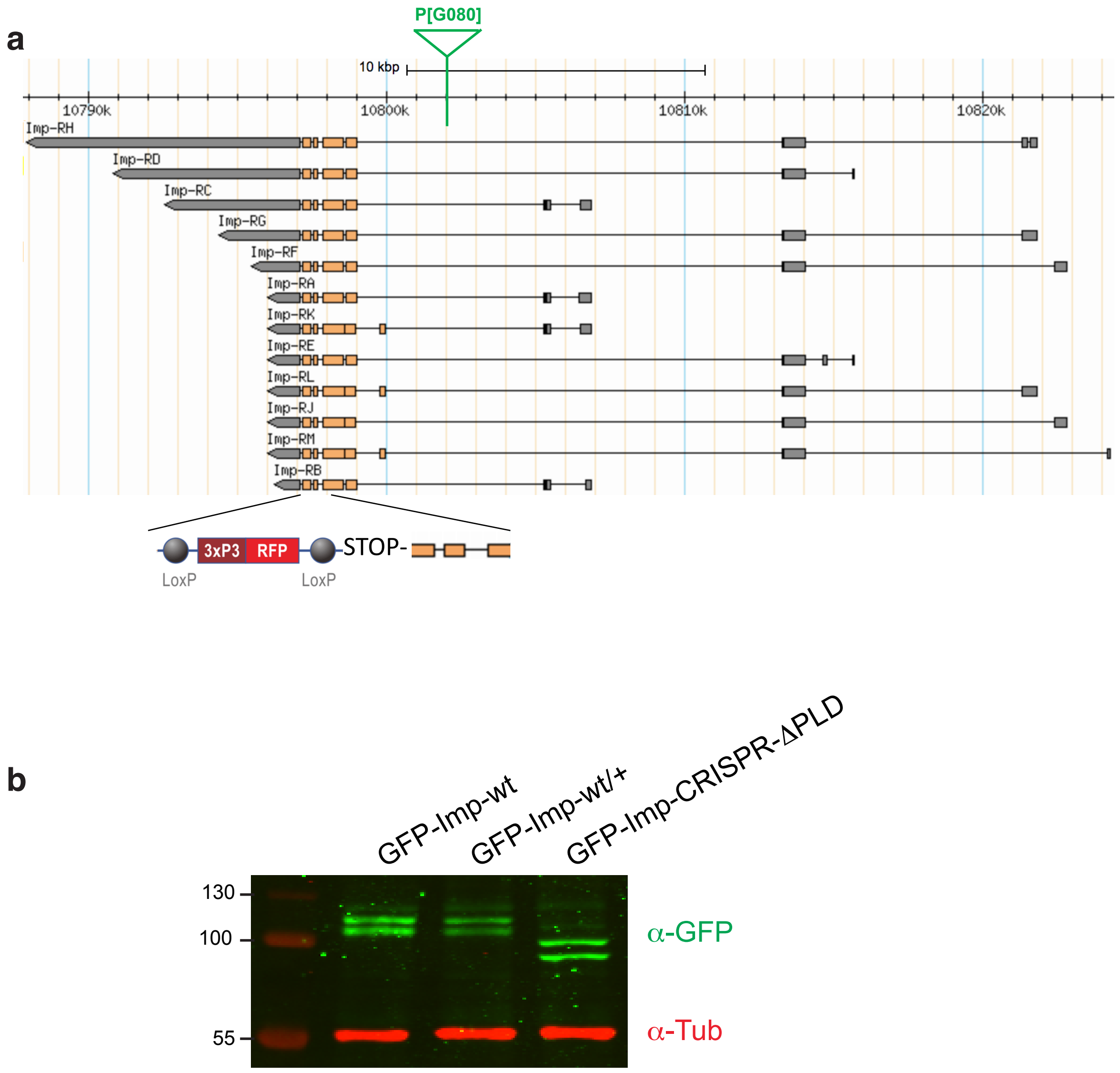

Supplementary Fig. 1 Description of the GFP-Imp-CRISPR-ΔPLD line. **a** Map of the *imp* genomic locus showing the G080 protein-trap insertion (top) together with the position of the CRISPR STOP cassette (bottom) (adapted from FlyBase). **b** Western-Blot of protein fractions recovered from heads homozygous (left) or heterozygous (middle) for the G080-GFP-Imp protein-trap insertion, and heads homozygous for the G080-GFP-Imp-CRISPR-ΔPLD chromosome (right). Proteins were stained with both anti-GFP (green) and anti-Tubulin (red) antibodies.

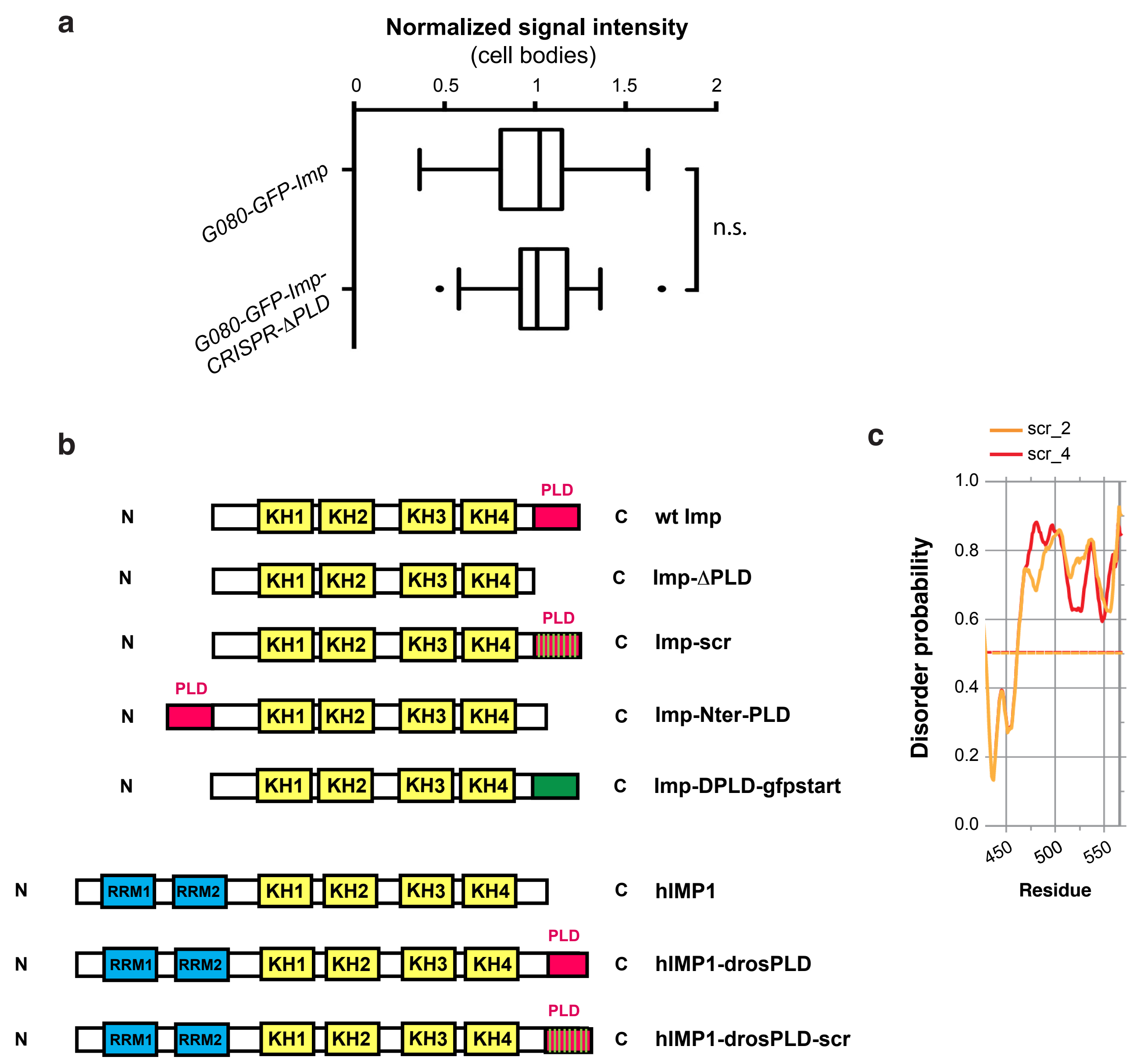

Supplementary Fig. 2 Imp variants. **a** Distributions of normalized GFP signal intensities in MB γ neuron cell bodies (Tukey box plots). n.s. stands for not significant. Source data are provided as a Source Data file. **b** Schematic representation of the different variants used in this study. **c** Plot of the degree of disorder along the Imp C-terminal region of Imp-scr2 and Imp-scr4, as predicted by the DisEMBL Intrinsic Protein Disorder Prediction 1.5 algorithm. Both variants have a predicted disordered C-terminal region.

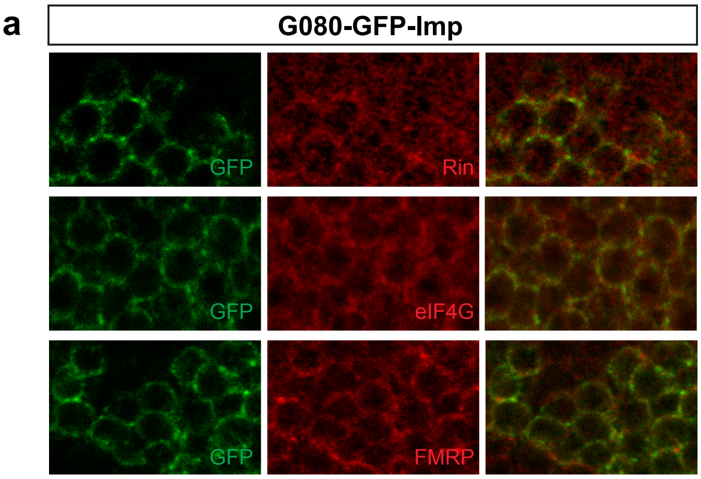

b

	presence in Imp granules ?		presence of a PLD?
	Imp-wt	Imp-ΔPLD	
Rin/GBP43	-	-	nd
eIF4G	-	-	nd
Rpl32	-	-	nd
FMRP	-	-	nd
eIF4e	+	+	no
RpS6	+/-	+/-	no
Pur-α	+	+	no
Staufen	+	+	yes
Me31B	+	+	no
GW182	+	+	no
Tral	+	+	yes

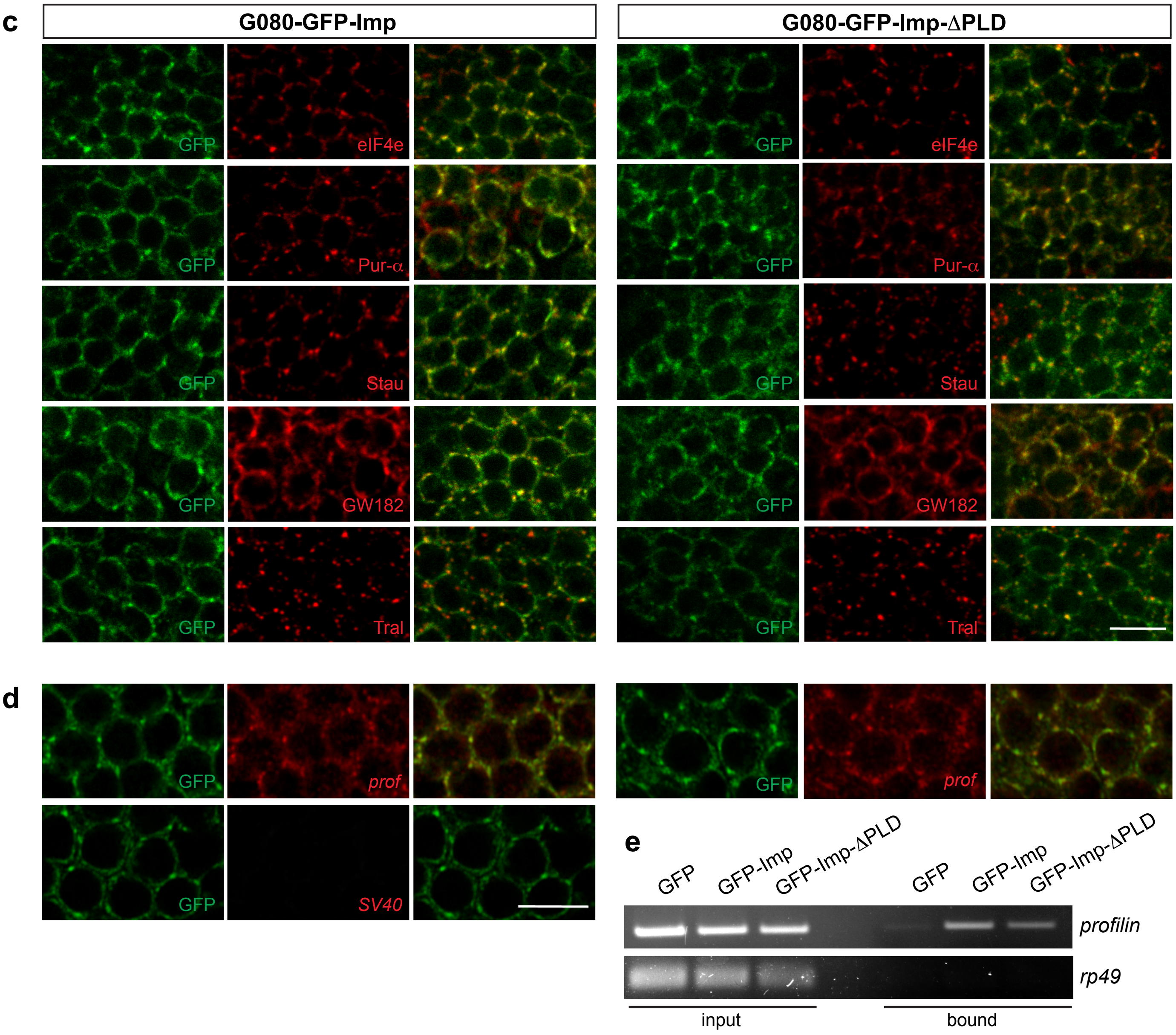

Supplementary Fig. 3 Composition of Imp granules. **a,c** Cell bodies of adult G080-GFP-Imp MB γ neurons stained with anti-GFP antibodies (green) and anti-Rin/G3BP (top), eIF4G (middle) or FMRP (bottom) antibodies (red). **b** Table summarizing the distribution of analyzed granule markers. Proteins contained in Imp granules were tested for the presence of a PLD or an intrinsically disorder region (IDR) using the softwares described in [1] and [2] respectively. **c** Cell bodies of adult G080-GFP-Imp (left) or G080-GFP-Imp-CRISPR-ΔPLD (right) MB γ neurons stained with anti-GFP antibodies (green) and anti-eIF4e, Pur-α, Staufen (Stau), GW182 or Tral antibodies (red). Scale bar in **a,c**: 5 μm. **d** Cell bodies of adult G080-GFP-Imp (left) or G080-GFP-Imp-CRISPR-ΔPLD (right) MB γ neurons stained with anti-GFP antibodies (green), and hybridized with stellaris probes complementary to *profilin* (*prof*) or *SV40* transcripts (red). Although *profilin* mRNA can be found outside Imp granules, a significant proportion of Imp granules contain *profilin* in both G080-GFP-Imp and G080-GFP-Imp-CRISPR-ΔPLD conditions. Scale bar in **d**: 5 μm. **e** Semi-quantitative RT-PCR amplifications of mRNAs recovered in fractions immunoprecipitated from 201Y-Gal4/UAS-GFP (GFP), 201Y-Gal4/UAS-GFP-Imp (GFP-Imp) or 201Y-Gal4/UAS-GFP-Imp-ΔPLD (GFP-Imp-ΔPLD) head extracts. *rp49* is used as a negative control.

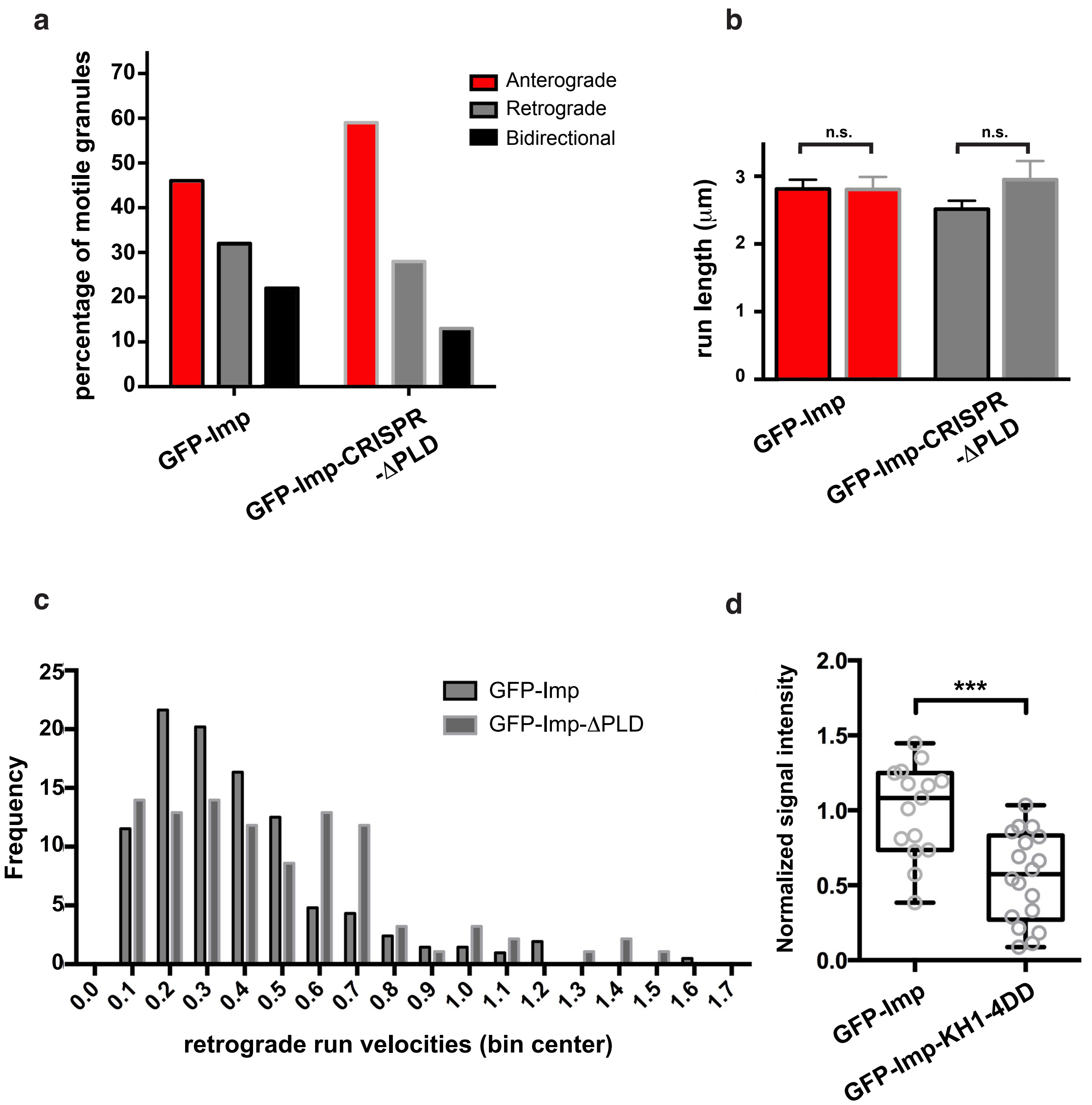

Supplementary Fig. 4 Characteristics of GFP-Imp granule motility. **a** Percentage of anterograde, retrograde and bidirectional granules. Total numbers of granules analyzed: 468 for G080-GFP-Imp and 284 for G080-GFP-Imp-CRISPR-Δ PLD. **b** Average lengths of anterograde (red) and retrograde (black) runs. Numbers of anterograde runs analyzed: 286 (G080-GFP-Imp) and 189 (G080-GFP-Imp-CRISPR-ΔPLD); numbers of retrograde runs analyzed: 208 (G080-GFP-Imp) and 93 (G080-GFP-Imp-CRISPR-ΔPLD). Error bars represent sem. ns stands for not significant. **c** Frequency distribution of retrograde run velocity values. **d** Distributions of normalized GFP signal intensities in distal axons. The box plot is represented using the min to max convention, where the middle line defines the median and the whiskers go down to the smallest value and up to the largest. Note that because GFP-KH1-4DD proteins were expressed at lower levels than GFP-Imp proteins, a higher laser power was used to image the GFP-KH1-4DD condition. Numbers of MBs analyzed: UAS-GFP-Imp: 15; UAS-GFP-Imp-KH1-4DD: 18. ***, $P < 0.001$ (Mann-Whitney test). Source data are provided as a Source Data file.

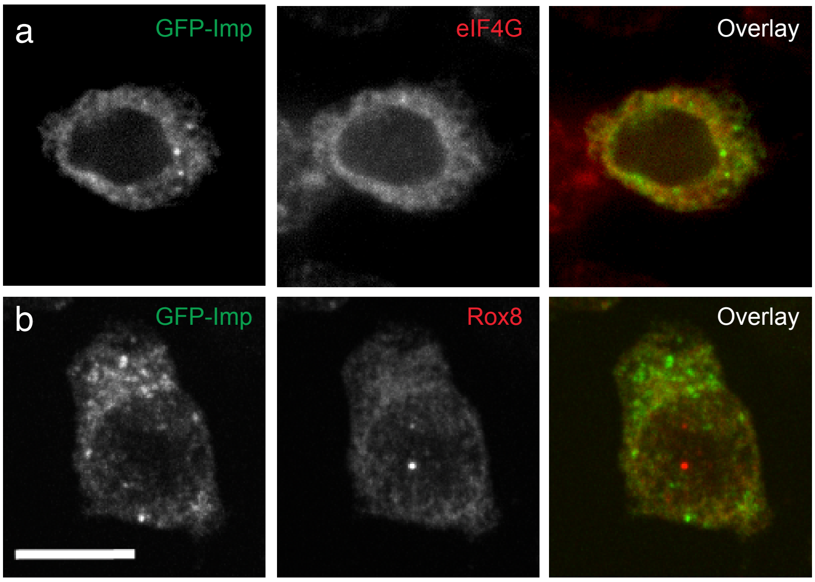

Supplementary Fig. 5 Imp RNP granules are distinct from stress granules. **a** S2R+ cells transfected with GFP-Imp (left, green in the overlay) and stained with anti-eiF4G antibodies (middle, red in the overlay). **b** S2R+ cell co-transfected with GFP-Imp (left, green in the overlay), and RFP-Rox8 (middle, red in the overlay). Rox8 is the fly orthologue of Tia-1. Scale bar: 10 μm.

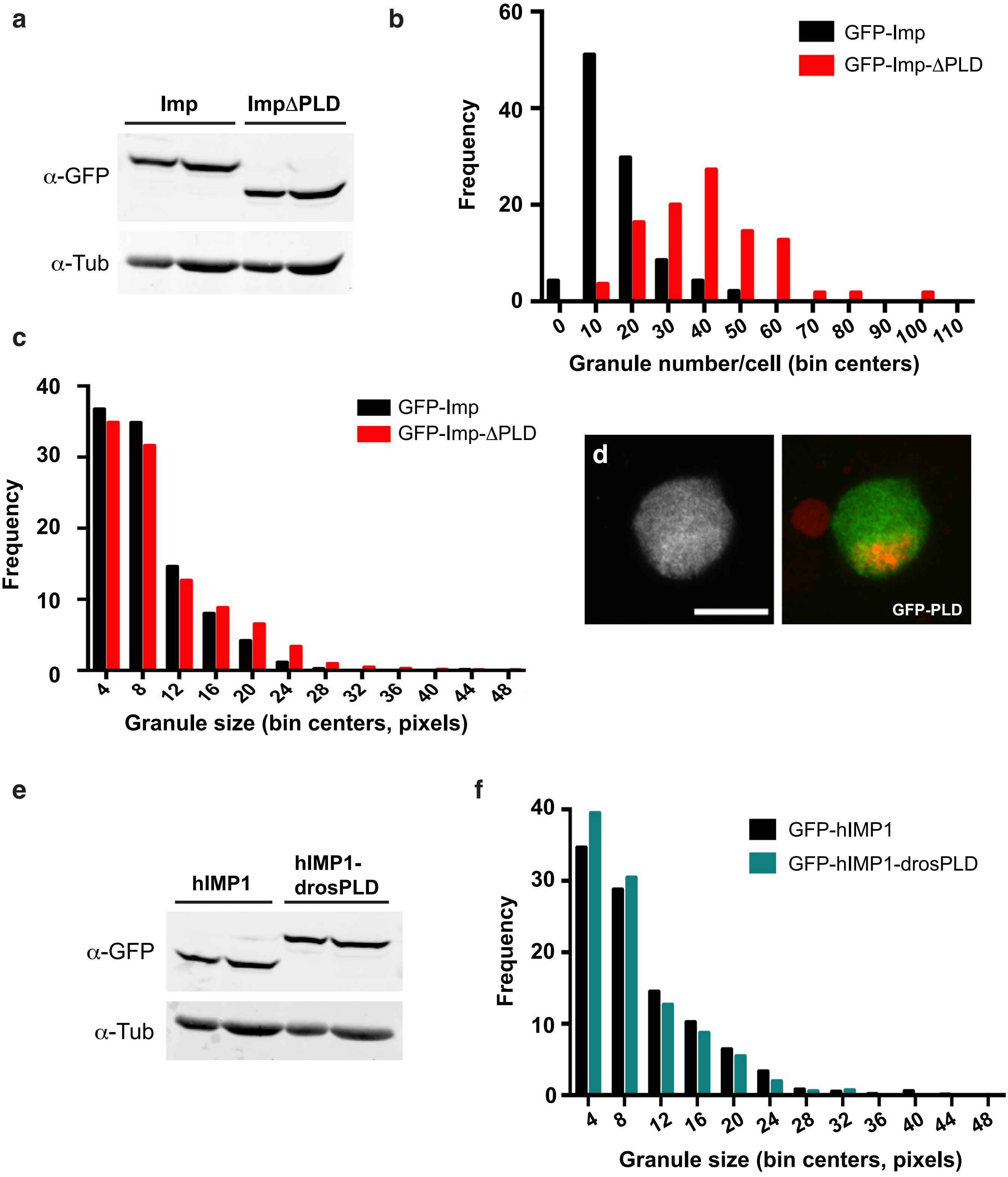

Supplementary Fig. 6 Imp PLD modulates granule properties. **a** Western-Blot performed on extracts from S2R+ cells transfected with GFP-Imp (left) or GFP-Imp-ΔPLD (right) constructs, using anti-GFP and anti-Tubulin antibodies. Two different amounts were loaded for each construct. **b** Frequency distributions of the number of GFP-Imp (black) and GFP-Imp-ΔPLD (red) granules per cell. **c** Frequency distributions of the size of GFP-Imp (black) and GFP-Imp-ΔPLD (red) granules. **d** S2R+ cell transfected with GFP-drosPLD only (left, green in the overlay), and stained with DAPI (red in the overlay). Scale bar: 10 µm. **e** Western-Blot performed on extracts from S2R+ cells transfected with GFP-hIMP1 (left) and GFP-hIMP1-drosPLD (right) constructs, using anti-GFP and anti-Tubulin antibodies. Two different amounts were loaded for each construct. **f** Frequency distributions of the size of GFP-hIMP1 (black) and GFP-hIMP1-drosPLD (green) granules. Source data are provided as a Source Data file.

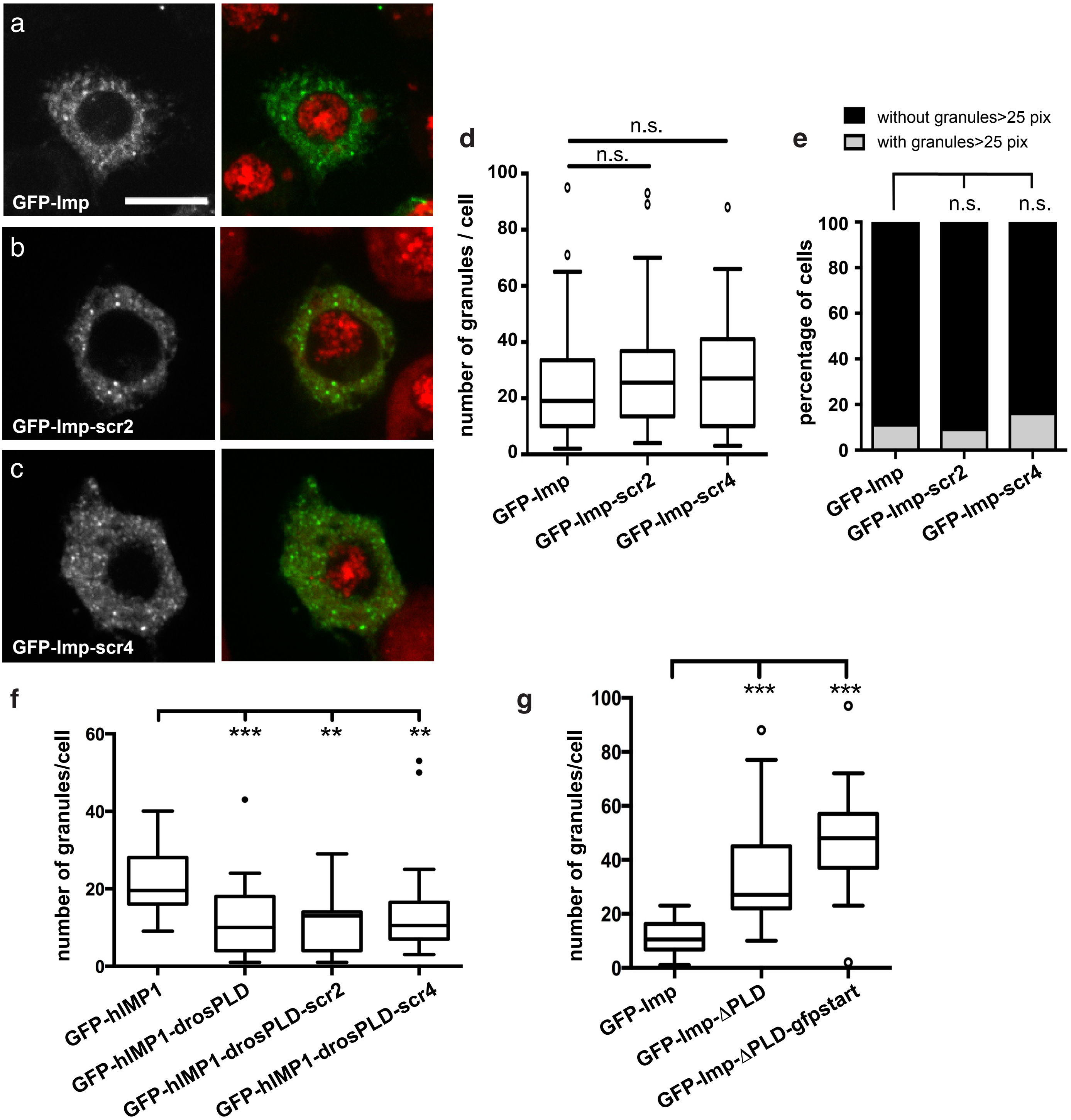

Supplementary Fig. 7 Molecular determinants underlying the function of Imp PLD in the regulation of Imp granule assembly. **a-c** S2R+ cells transfected with GFP-Imp (**a**), GFP-Imp-scr2 (**b**) or GFP-Imp-scr4 (**c**) constructs, and stained with DAPI (red in the overlay). GFP signals are shown in white (left) and green in the overlay. Scale bar: 10 µm. **d** Distribution of cells in function of their number of granules (Tukey box plots). **e** Percentage of cells exhibiting granules larger than 25 pixels. ns stands for not significant. 65, 80 and 62 cells were analyzed for GFP-Imp, GFP-Imp-scr2 and GFP-Imp-scr4 respectively. **f** Distribution of cells in function of their number of granules (Tukey box plots). S2R+ cells were transfected with GFP-hIMP1, GFP-hIMP1-drosPLD, GFP-hIMP1-drosPLDscr2, or GFP-hIMP1-drosPLDscr4. 30, 23, 19 and 24 cells were analyzed respectively. **, $P<0.01$; ***, $P<0.001$ (One-way ANOVA with Dunnett's multiple comparison tests). **g** Distribution of cells in function of their number of granules (Tukey box plots). S2R+ cells were transfected with GFP-Imp, GFP-Imp-ΔPLD and GFP-Imp-ΔPLD-gfpstart (construct where the first 300 bp of GFP have been added C-terminally to Imp-ΔPLD). 26, 31 and 32 cells were analyzed respectively. **, $P<0.01$ (One-way ANOVA with Dunnett's multiple comparison tests). Source data are provided as a Source Data file.

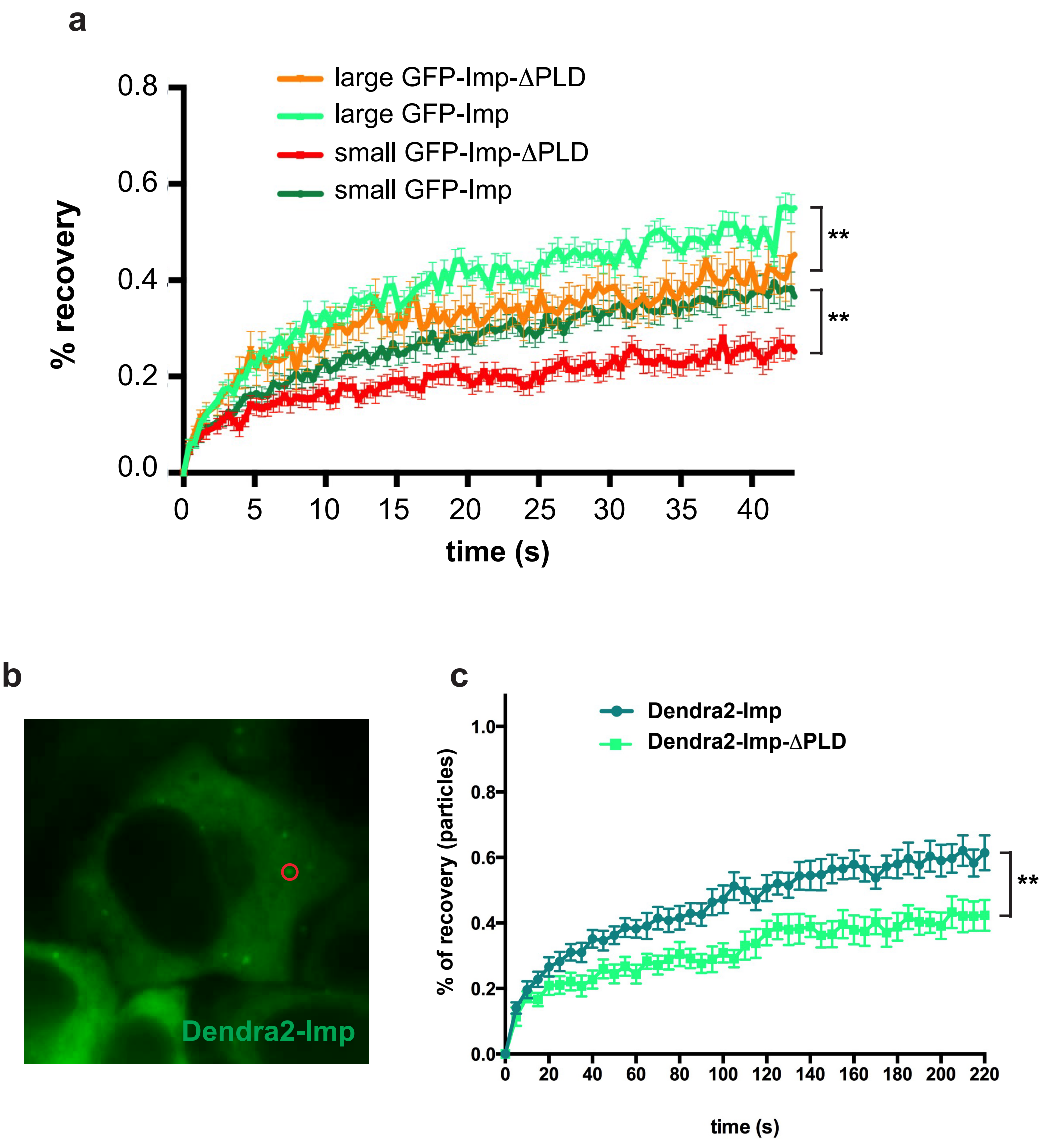

Supplementary Fig. 8 Imp PLD regulates the exchange of Imp in and out granules in HeLa cells. **a** Average FRAP curves obtained after photobleaching of small (approximately 5 pixels) or large (9 pixel diameter) GFP-positive particles from S2R+ cells. The following numbers of particles were analyzed: large GFP-Imp: 44; large GFP-Imp-ΔPLD: 40; small GFP-Imp: 40; small GFP-Imp-ΔPLD: 36. Error bars indicate s.e.m. **, $P < 0.01$ (Mann-Whitney test on the distributions of normalized intensity values at t= 45 s). **b** HeLa cell transfected with Dendra2-Imp (in green). The red circle delimits the photobleached region. **c** Average FRAP curves obtained after photobleaching of Dendra2-positive particles. The following numbers of particles were analyzed: Dendra2-Imp: 22; Dendra2-Imp-ΔPLD: 20. Error bars in b indicate s.e.m. **, $P < 0.01$ (Mann-Whitney test on the distributions of normalized intensity values at t= 220 s). Source data are provided as a Source Data file.

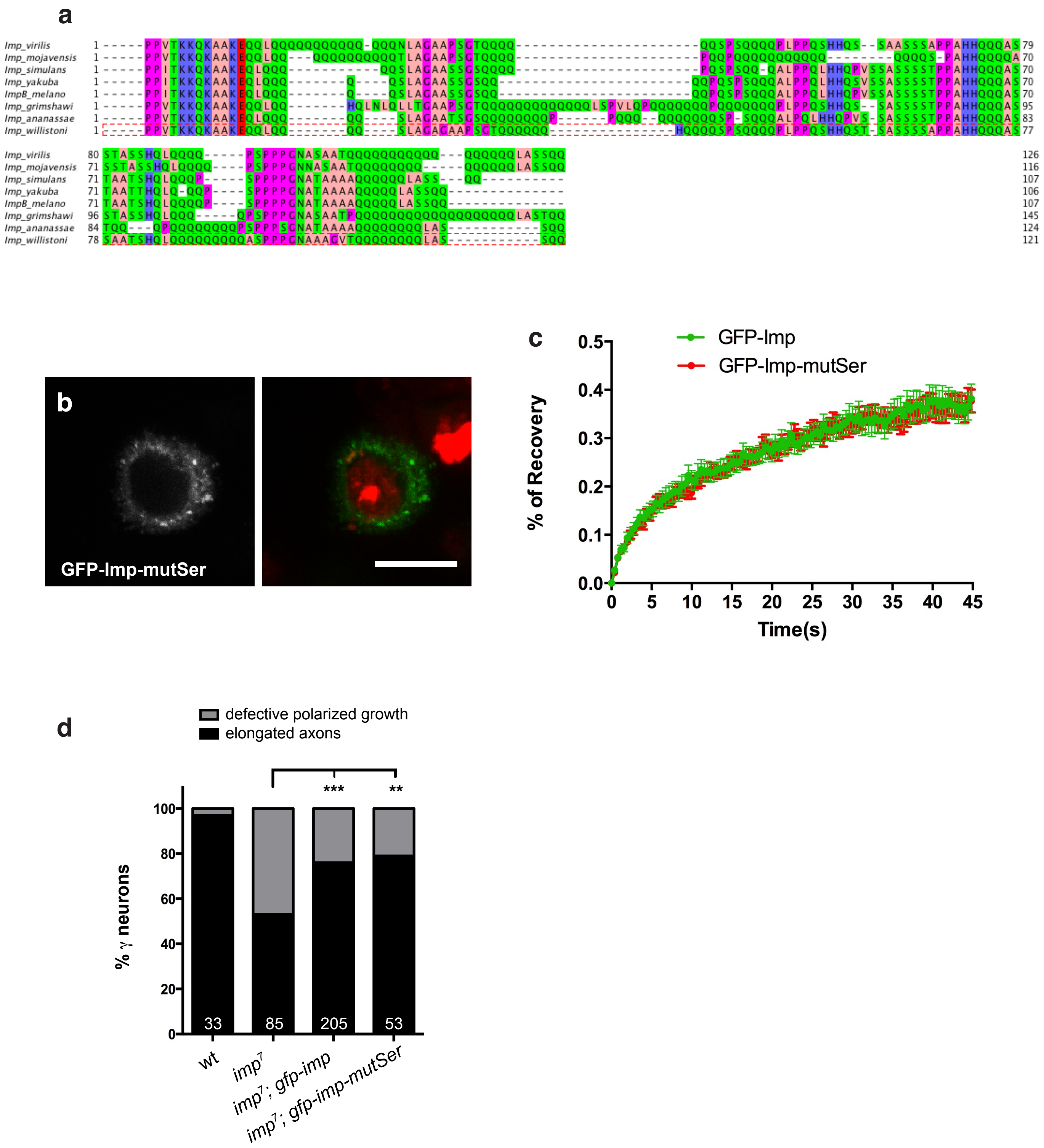

Supplementary Fig. 9 Features of Imp PLD sequence. **a** Alignment of Imp PLD from different *Drosophila* species. **b** S2R+ cell transfected with GFP-Imp-mutSer, and stained with DAPI (red in the overlay). In the Imp-mutSer construct, all Serines found in the PLD were mutated into Glycines. GFP signal is shown in white (left) and green in the overlay. Scale bar: 10 µm. **c** Average FRAP curves obtained after photobleaching of GFP-positive particles from S2R+ cells. The following numbers of particles were analyzed: GFP-Imp: 35; GFP-Imp-mutSer: 57. Error bars indicate s.e.m. **d** Percentages of adult γ axons that succeeded (elongated axon) or failed (defective axonal growth) to reach the extremity of the medial lobe. ***, $P < 0.001$; **, $P < 0.01$ (Fisher's exact test). Numbers correspond to the total numbers of scored individual axons.

Supplementary Table 1. Name and sequence of primers used to clone the different variants analyzed.

Name	Primer sequence (5' -> 3')	Comments
**impCS_sense**	CACC AGCAACAATAATAGC	used to generate the Imp-ΔPLD variant
**KH34-Gtwy rev**	TTACTGTTGCTGTTGTTGCAA	
**mut KH1 DD for**	ATGGTGGGCGCCATCATTGGTGATGACGGCAGCACCATCAG	used to generate the KH1-4DD variant
**mut KH1 DD rev**	GTGTGATCGTCCTGATGGTGCTGCCGTCATCACCAATGATGG	
**mut KH2 DD for**	TGATTGGACGAATCATTGGCGACGATGGCAATACCATTAAACG	
**mut KH2 DD rev**	ATGATCCGTTTAATAGTATTGCCATCGTCGCCAATGATTCGTC	
**mut KH3 DD for**	CAACGCTGTCGGCGCCATTATCGGCGACGACGGCTCGCATATCCG	
**mut KH3 DD rev**	GCATTATGCTTCGGATATGCGAGCCGTCGTCGCCGATAATGGCGC	
**mut KH4 DD for**	GTTCTCAGGTGGGCCGTATCATTGGCGACGATGGCCAAAATGTGC	
**mut KH4 DD rev**	CTGCAATTCCCGCACATTTTGGCCATCGTCGCCAATGATACGGCC	
**hIMP1-Gtwy for**	CACCATGAACAAGCTTTACATCGG	used to generate the hIMP1 and hIMP1-drosPLD variants
**hIMP1-Gtwy-RP**	TTATCACTTCCTCCGTGCCTGG	
**hIMP1 + Imp PLD rev**	CAGGCACGGAGGAAGAGCCTAGCCGGAGC	
**hIMP1 + Imp PLD FD**	GCTCCGGCTAGGCTCTTCCTCCGTGCCTG	
**Imp PLD rev**	TCCTTTAACGGTGATTCC	
**Imp_just before PLD + Scr V2 rev**	CTGTGCCTGCAGCTGTTGCTGT	used to generate the Imp-scr2 variant
**Imp + Scr V2 for**	CAACAGCAACAGCTGCAGGCACAG	
**Imp + Scr V2 rev**	TTATGACGGGCCTTGGCTAGC	
**Imp_just before PLD + Scr V4 rev**	CTGGGCTTGGCTCTGTTGCTGTTG	used to generate the Imp-scr4 variant
**Imp + Scr V4 for**	CAACAGCAACAGAGCCAAGCCCAG	
**Imp + Scr V2 rev**	TTAAGGTGGGCTTTGCAACTG	
**PLD Gtway for**	CACCATGAGCCTAGCCGGAGCCG	used to generate the Imp-Nter-PLD variant
**PLD ImpB rev**	TTATTGTTGCTGTGCATCTGTTGTGAGCTCGCCAGC	
**PLD ImpB for**	GCTGGCGAGCTCACAACAGATGCACAGCAACAATAA	
**PLD Gtway for**	CACCATGAGCCTAGCCGGAGCCG	used to generate the drosPLD construct
**impCS_antisense**	TCCTTTAACGGTGATTCC	
**ImpBDQ+GFP fd**	GCAACAACAGCAACAGATGGTGAGCAAGG	used to generate the Imp-ΔPLD-gfpstart variant
**ImpBDQ+GFP rev**	CCTTGCTCACCATCTGTTGCTGTTGTTGC	
**hImp + V2 rev:**	CTGTGCCTGCAGCTTCCTCCGTGCCTGG	used to generate the hIMP1-drosPLD-scr variant
**hImp + Puc 57 Scramble V2 fd**	CCAGGCACGGAGGAAGCTGCAGGCACAG	
**hImp+ Puc 57 Scramble V4 rev**	CTGGGCTTGGCTCTTCCTCCGTGCCTGG	
**hImp+ Puc 57 Scramble V4 fd**	CCAGGCACGGAGGAAGAGCCAAGCCCAG	

Supplementary Table 1. Name and sequence of primers used to clone the different variants analyzed.

Supplementary references

1- Malinovska, L., Kroschwald, S. & Alberti, S. Protein disorder, prion propensities, and self-organizing macromolecular collectives. *Biochim Biophys Acta* **1834**, 918-931, doi:10.1016/j.bbapap.2013.01.003 (2013).

2- Jones, D. T. & Cozzetto, D. DISOPRED3: precise disordered region predictions with annotated protein-binding activity. *Bioinformatics* **31**, 857-863, doi:10.1093/bioinformatics/btu744 (2015).
