# Supplementary material for: The prion-like domain of Drosophila Imp promotes axonal transport of RNP granules in vivo
Source: Nat Commun. 2019 Jun 13;10:2593. doi: 10.1038/s41467-019-10554-w (PMC6565635; doi:10.1038/s41467-019-10554-w)
Supplement: Supplementary file 3 — Description of Additional Supplementary Files [file 41467_2019_10554_MOESM3_ESM.pdf]

Description of Additional Supplementary Files

File Name: Supplementary Movie 1
Description: Real-time imaging of endogenous GFP-Imp granules. G080-GFP-Imp brain imaged 24h After Puparium Formation (APF) in the proximal part of MB g axons (peduncle). Anterograde granules move from left to right and retrograde ones from right to left. Images were acquired every 1.2 s. Scale bar: 10 µm.
