# Supplementary material for: The prion-like domain of Drosophila Imp promotes axonal transport of RNP granules in vivo
Source: Nat Commun. 2019 Jun 13;10:2593. doi: 10.1038/s41467-019-10554-w (PMC6565635; doi:10.1038/s41467-019-10554-w)
Supplement: Supplementary file 5 — Reporting Summary [file 41467_2019_10554_MOESM5_ESM.pdf]

nature research

Corresponding author(s): Florence Besse

Last updated by author(s): 2019/01/11

Reporting Summary

Nature Research wishes to improve the reproducibility of the work that we publish. This form provides structure for consistency and transparency in reporting. For further information on Nature Research policies, see Authors & Referees and the Editorial Policy Checklist.

Statistics

For all statistical analyses, confirm that the following items are present in the figure legend, table legend, main text, or Methods section.

n/a	Confirmed	
☐	☒	The exact sample size (n) for each experimental group/condition, given as a discrete number and unit of measurement
☐	☒	A statement on whether measurements were taken from distinct samples or whether the same sample was measured repeatedly
☐	☒	The statistical test(s) used AND whether they are one- or two-sided<br>*Only common tests should be described solely by name; describe more complex techniques in the Methods section.*
☐	☒	A description of all covariates tested
☐	☒	A description of any assumptions or corrections, such as tests of normality and adjustment for multiple comparisons
☐	☒	A full description of the statistical parameters including central tendency (e.g. means) or other basic estimates (e.g. regression coefficient) AND variation (e.g. standard deviation) or associated estimates of uncertainty (e.g. confidence intervals)
☒	☐	For null hypothesis testing, the test statistic (e.g. F, t, r) with confidence intervals, effect sizes, degrees of freedom and P value noted<br>*Give P values as exact values whenever suitable.*
☒	☐	For Bayesian analysis, information on the choice of priors and Markov chain Monte Carlo settings
☒	☐	For hierarchical and complex designs, identification of the appropriate level for tests and full reporting of outcomes
☒	☐	Estimates of effect sizes (e.g. Cohen's d, Pearson's r), indicating how they were calculated

Our web collection on statistics for biologists contains articles on many of the points above.

Software and code

Policy information about availability of computer code

Data collection	n/a
Data analysis	The SPADE algorithm (https://raweb.inria.fr/rapportsactivite/RA2016/morpheme/uid13.html) was used for quantification of granule properties.

For manuscripts utilizing custom algorithms or software that are central to the research but not yet described in published literature, software must be made available to editors/reviewers. We strongly encourage code deposition in a community repository (e.g. GitHub). See the Nature Research guidelines for submitting code & software for further information.

Data

Policy information about availability of data

All manuscripts must include a data availability statement. This statement should provide the following information, where applicable:

- Accession codes, unique identifiers, or web links for publicly available datasets
- A list of figures that have associated raw data
- A description of any restrictions on data availability

All relevant data are available from the corresponding author upon reasonable request.

Field-specific reporting

Please select the one below that is the best fit for your research. If you are not sure, read the appropriate sections before making your selection.

☒ Life sciences ☐ Behavioural & social sciences ☐ Ecological, evolutionary & environmental sciences

Life sciences study design

All studies must disclose on these points even when the disclosure is negative.

Sample size	Sample sizes (for cells and flies) were initially determined empirically, but we verified that the power of the statistal tests used was >80% for each representative set of experiments.
Data exclusions	No data were excluded from the analysis
Replication	All data presented here are representative of at least two independent experiments. Graphs represent the results of at least two, mostly three replicates.
Randomization	n/a: flies were included into groups based on their genotypes. Cells were included into groups based on the identity of their transfected constructs.
Blinding	Samples were not blinded as quantifications relied on computational softwares (e.g. SPADE) or measure of signal intensities, avoiding any "subjective" assessments of phenotypes.

Reporting for specific materials, systems and methods

We require information from authors about some types of materials, experimental systems and methods used in many studies. Here, indicate whether each material, system or method listed is relevant to your study. If you are not sure if a list item applies to your research, read the appropriate section before selecting a response.

Materials & experimental systems

n/a	Involved in the study	
☐	☒	Antibodies
☐	☒	Eukaryotic cell lines
☒	☐	Palaeontology
☐	☒	Animals and other organisms
☒	☐	Human research participants
☒	☐	Clinical data

Methods

n/a	Involved in the study	
☒	☐	ChIP-seq
☒	☐	Flow cytometry
☒	☐	MRI-based neuroimaging

Antibodies

Antibodies used	- The following antibodies were used for Western-Blots: rabbit anti-GFP (1:2500; #TP-401: Torey Pines); mouse anti-Tubulin (1:5000 ; DM1A clone; Sigma).<br>- The following antibodies were used for immuno-fluorescence: rat anti-Imp (1:1000; Medioni et al., 2014), rabbit anti-GFP (Molecular Probes, 1:1000), mouse anti-FasciclinII (DSHB, 1D4 clone; 1:15), rabbit anti-Rin (1:200, gift from E. Gavis), rabbit anti-eIF4G (1:1000, gift from E. Izaurralde), rabbit anti-Rpl32 (1:1000; gift from M. Henze); mouse anti-FMRP (DSHB, 2F5-1 clone, 1:50), rabbit anti-eIF4e (gift from E. Izaurralde), rat anti-Pur-α (1:50; gift from K. Förstemann), rat anti-Staufen (1:1000, gift from A. Ephrussi), rabbit anti-Me31B (1:500, gift from C. Lim), rabbit anti-Tral (1:1000, gift from A. Nakamura), rabbit anti-Gawky (1:1000; gift from E. Izaurralde).
Validation	Descriptions and validations of the Drosophila antibodies can be found on the DHSB website (http://dshb.biology.uiowa.edu/) or in the respective original publications:<br>- rat anti-Imp (Medioni et al., 2014; DOI:10.1016/j.cub.2014.02.038)<br>- rabbit anti-Rin (Aguilera-Gomez et al., 2017; DOI: 10.1016/j.celrep.2017.06.042)<br>- rabbit anti-eIF4G and eIF4e (Zekri et al., 2013; DOI:10.1038/emboj.2013.44)<br>- rat anti Pur-a (Aumiller et al., 2012; DOI:10.4161/rna.19760)<br>- rat anti-Staufen (Ghosh et al., 2014; DOI:10.1371/journal.pgen.1004455)<br>- rabbit anti-Me31B (Lee et al., 2017; DOI:10.1016/j.molcel.2017.03.004)<br>- rabbit anti-Gawky (Behm-Ansmant et al., 2006; DOI:10.1101/gad.1424106

Eukaryotic cell lines

Policy information about cell lines

Cell line source(s)	Drosophila Genomics Resource Center (https://dgrc.bio.indiana.edu/Home)
Authentication	S2R+ cells received from the DGRC were not authenticated.
Mycoplasma contamination	S2R+ cells were not tested for Mycoplasma contamination.

Commonly misidentified lines (See ICLAC register)	n/a

Animals and other organisms

Policy information about studies involving animals; ARRIVE guidelines recommended for reporting animal research

Laboratory animals	w1118-derived Drosophila melanogaster individuals were used in our study. Unless specified, males and females were indistinguishly used. 4-6 day old-flies were dissected.
Wild animals	n/a
Field-collected samples	n/a
Ethics oversight	n/a

Note that full information on the approval of the study protocol must also be provided in the manuscript.
